# Geriatric oncology in Spain: survey results and analysis of the current situation

**DOI:** 10.1007/s12094-017-1813-0

**Published:** 2017-12-11

**Authors:** R. Gironés, I. Morilla, C. Guillen-Ponce, M. D. Torregrosa, I. Paredero, E. Bustamante, S. del Barco, G. Soler, B. Losada, L. Visa, E. Llabrés, B. Fox, J. L. Firvida, R. Blanco, M. Antonio, F. Aparisi, M. Pi-Figueras, E. Gonzalez-Flores, M. J. Molina-Garrido, J. Saldaña, On behalf of the Spanish Working Group on Geriatric Oncology of the Spanish Society of Medical Oncology (SEOM)

**Affiliations:** 1 Medical Oncology Unit. Hospital Lluís Alcanyís, Crta Xàtiva A Silla Km 2, Xàtiva, 46800 Valencia, Spain; 2 Institut Català D’Oncologia-L’Hospitalet, Barcelona, Spain; 3 Hospital Universitario Ramón Y Cajal, Madrid, Spain; 4 Hospital Universitario Dr Peset, Valencia, Spain; 5 Althaia, Xarxa Assistencial I Universitaria Manresa, Barcelona, Spain; 6 Hospital Universitari Dr. Josep Trueta. ICO Girona, Girona, Spain; 7 Hospital Universitario de Fuenlabrada, Madrid, Spain; 8 Hospital Del Mar, Barcelona, Spain; 9 Hospital Universitario Insular de Gran Canaria, Las Palmas, Spain; 10 Hospital General Universitario Gregorio Marañón, Madrid, Spain; 11 Complejo Hospitalario Universitario de Ourense (CHUO), Ourense, Spain; 12 Consorci Sanitari de Terrassa, Barcelona, Spain; 13 Hospital General de Valencia, Valencia, Spain; 14 Hospital Virgen de Las Nieves, Granada, Spain; 15 Hospital General Virgen de La Luz, Cuenca, Spain

**Keywords:** Geriatric oncology, Spanish survey, Spanish Society of Medical Oncology (SEOM), Spanish Working Group in Geriatric Oncology

## Abstract

**Introduction:**

Geriatric oncology (GO) is a discipline that focuses on the management of elderly patients with cancer. The Spanish Society of Medical Oncology (SEOM) created a Working group dedicated to geriatric oncology in February 2016.

**Objectives:**

The main goal of this study was to describe the current situation in Spain regarding the management of elderly cancer patients through an online survey of medical oncologists.

**Methods:**

A descriptive survey was sent to several hospitals by means of the SEOM website. A personal e-mail was also sent to SEOM members.

**Results:**

Between March 2016 and April 2017, 154 answers were collected. Only 74 centers (48%) had a geriatrics department and a mere 21 (14%) medical oncology departments had a person dedicated to GO. The vast majority (*n* = 135; 88%) had the perception that the number of elderly patients with cancer seen in clinical practice had increased. Eighteen (12%) oncologists had specific protocols and geriatric scales were used at 55 (31%) centers. Almost all (92%) claimed to apply special management practices using specific tools. There was agreement that GO afforded certain potential advantages. Finally, 99% of the oncologists surveyed believed it and that training in GO had to be improved.

**Conclusions:**

From the nationwide survey promoted by the Spanish Geriatric Oncology Working Group on behalf of SEOM, we conclude that there is currently no defined care structure for elderly cancer patients. There is an increasing perception of the need for training in GO. This survey reflects a reality in which specific needs are perceived.

## Introduction

Geriatric oncology is a field of medicine that has advanced tremendously. The International Society of Geriatric Oncology (SIOG) [1] has joined forces to implement geriatric oncology in clinical practice worldwide.

To translate SIOG efforts to our country (Spain), the Geriatric Oncology Working Group of the Spanish Medical Oncology Society (SEOM) was created on 29 February, 2016. This group consists of 50 members, most of whom are medical oncologists.

One of the Working Group’s proposals was to analyze oncologists’ current status, needs, and perceptions, as regards the management of the elderly in our country. The work was approved by the Spanish Society of Medical Oncology (SEOM).

## Materials and methods

The survey was a closed, 10-question, online questionnaire (Table 1). The first two questions had to do with the specialties that provide hospital care for elderly patients. The next two questions were addressed to medical oncologists, asking about specific management practices or protocols within their discipline. The last five questions appraised oncologists’ perceptions, needs, and the use of geriatric assessments.

**Table 1:** Online questionnaire

1. At your center, what disciplines are involved in geriatric care? (list)
2. Of those disciplines, which are involved in geriatric oncology care (list)
3. In medical oncology, is there any physician at your institution in charge of geriatric oncology? Yes. No. All of us
4. Are there guidelines for geriatric oncology at your institution? Yes. No
5. Do you perceive an increase in elderly patients in clinical practice? Yes. No. I have not thought about it
6. Do you believe that care for elderly should differ from regular adult care? Yes. No. I have not thought about it
7. Do you feel that you need scales other than performance status to care for this population? Yes. No
8. Do you use any geriatric scale for seniors with cancer in clinical practice?
9. What do you feel geriatric oncology should bring to oncology? (list)
10. Do you feel that you need more information or education in geriatric oncology?

To save time, the survey purposely did not include possible recommendations, thereby facilitating rapid compilation. The questionnaire was anonymous and did not collect personal date, such as gender, age, type of hospital, or cancer type.

From March 2016 to April 2017, the survey was accessible on the SEOM website. To motivate SEOM members, a personal e-mail was also sent to increase participation.

### Statistical analysis

Qualitative variables were reported as numbers (*N*) and percentages. Statistical analyses were performed using *χ* ^2^ or Fisher’s exact test. Results were considered statistically significant with *p* < 0.05. Statistical analyses were conducted using Stata software (version 11).

## Results

**Fig. 1:**
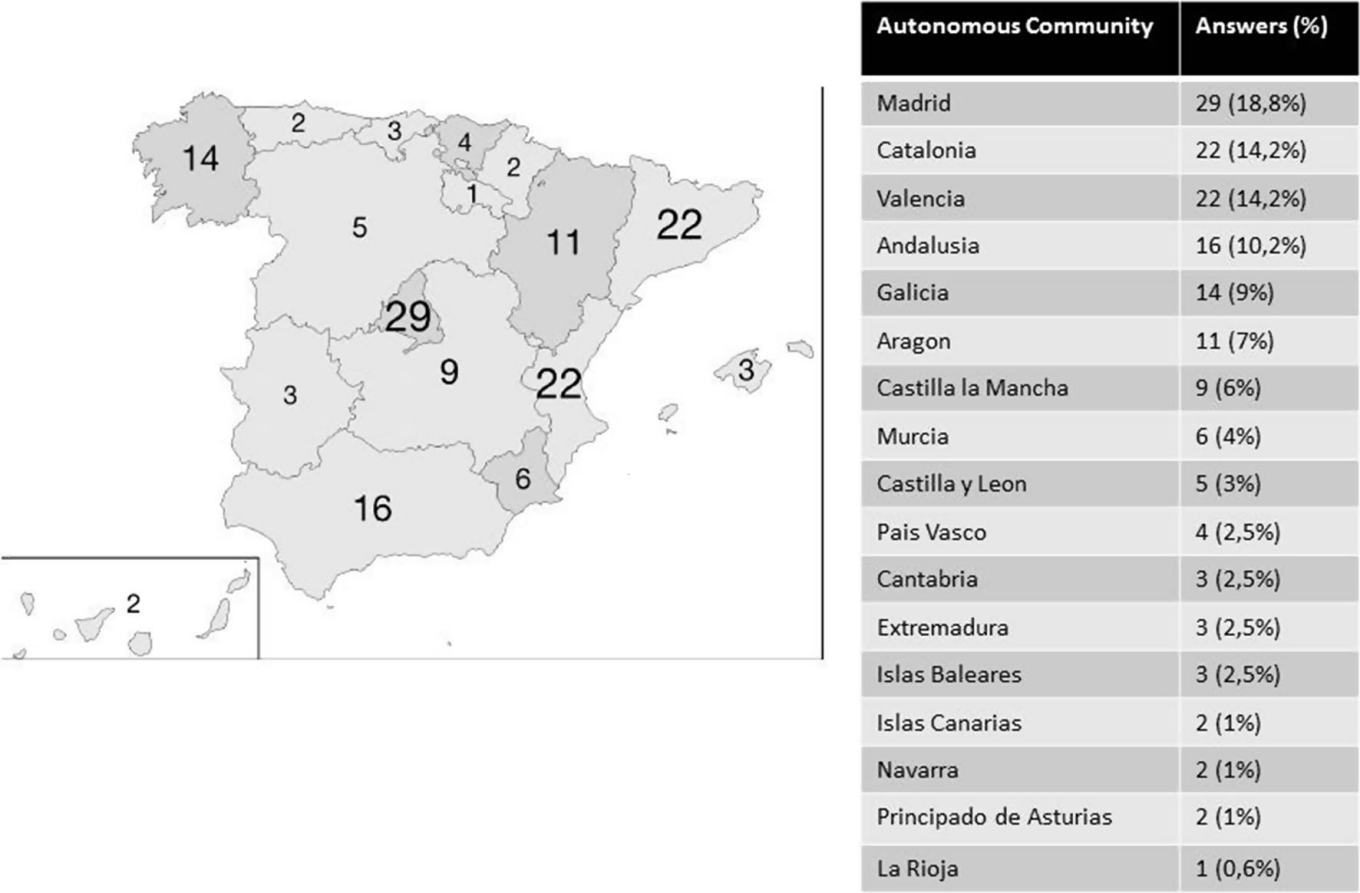
Answers by the autonomous community

The survey was answered by 154 medical oncologists from 154 centers, with good geographical representation of the medical oncology departments in Spain (Fig. 1).

**Fig. 2:**
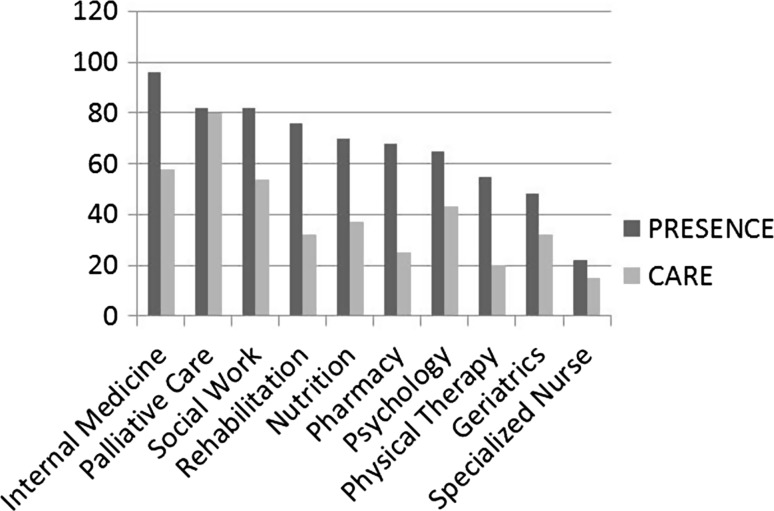
Prevalence of specialties and percentage that focus on older patients with cancer at Spanish hospitals

The first question referred to the specialties at the centers that cared for elderly patients (not only those with cancer). At most centers, internal medicine (148; 96.1%) and palliative care (127; 82%) cared for the elderly population. However, fewer than half of the centers (75; 48.5%) had a geriatrics discipline (Fig. 2).

When we asked whether the disciplines mentioned cared for seniors with cancer or not, palliative care ranked first (123; 80%), followed by internal medicine (90; 58%); geriatrics was in charge of caring for these patients in a lower percentage (50; 32%).

**Fig. 3:**
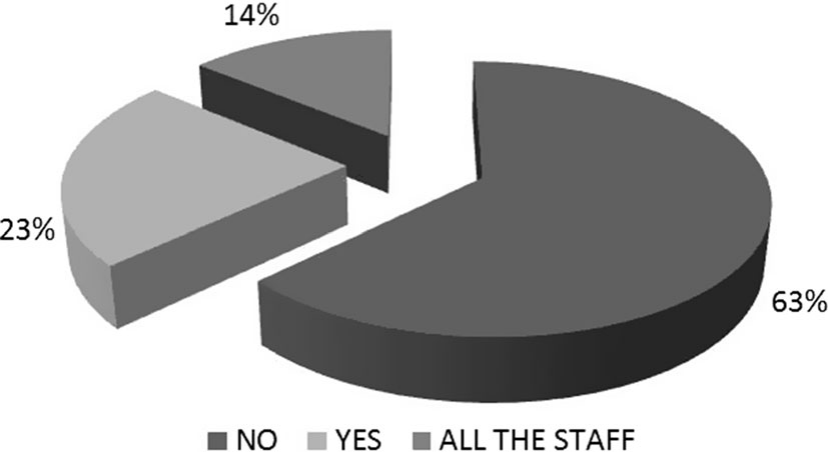
Is there a department or a unit dedicated to seniors within the oncology department where you work?

When asked if there was a physician who specialized in elderly oncological patient management at their department, only 22 (14%) answered in the affirmative. The remaining 35 (23%) oncologists were in charge of caring for the elderly, with no one person in particular in charge. Most centers (97; 63%) acknowledged the absence of a geriatric oncology specialist (Fig. 3).

Almost all of the respondents stated that their centers lacked institutional guidelines for elderly cancer patients (135; 88%). Only 10 (7%) centers reported having specific guidelines for certain types of tumors. Most (nearly 80%) of the responding medical oncologists perceived an increase in the number of seniors with cancer in their daily clinical practice. Only 11% responded that they did not perceive an increase in the prevalence of elderly patients at their services; 11% stated that they were not sure.

According to the opinion of most of the medical oncologists (93%), special care and additional attention should be provided for older patients in comparison to younger adults and a similar number (92%) also agreed that performance status did not suffice to evaluate elderly cancer patients.

When asked about the use of geriatric scales, 91 (59%) had never used them; 47 (31%) oncologists did use geriatric assessment in clinical practice, although in only 5% of the cases, geriatric assessment was conducted by a geriatrician. Finally, seven interviewees (5%) acknowledged that they had never heard about geriatric assessment.

**Fig. 4:**
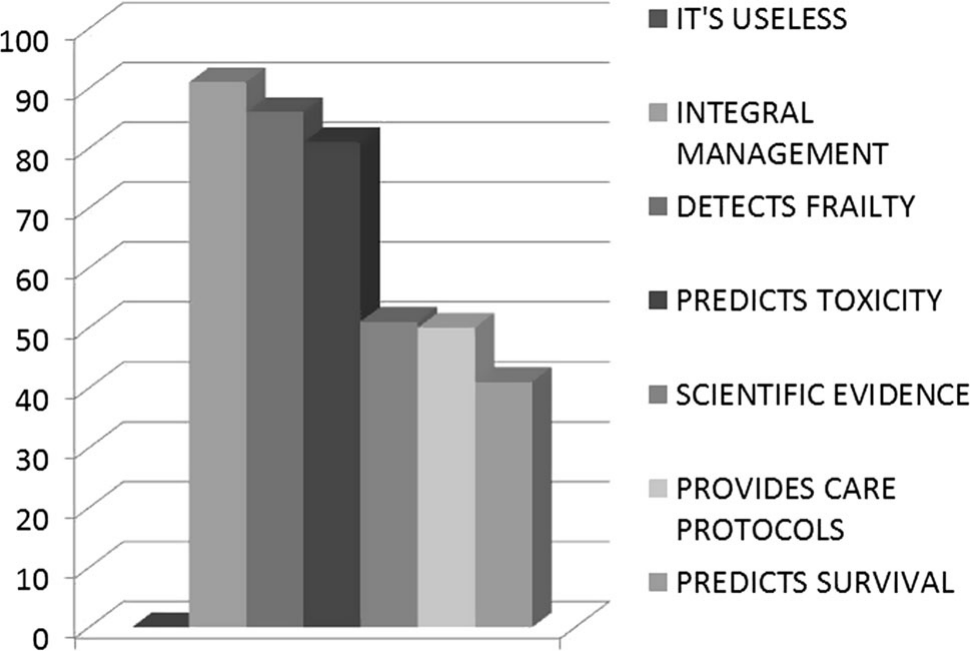
Oncologists’ perception of the usefulness of geriatric assessment

Finally, medical oncologists were asked about the usefulness of geriatric Assessment and why oncologists believed that geriatric oncology would help them (Fig. 4). Nearly all agreed that geriatric assessment was useful; 90% felt that it should be routinely administered to all the elderly patients; 80% believed that it could foresee treatment toxicity, and 85% considered that it enables frailty to be detected.

All the respondents agreed that they needed more training in geriatric oncology (100%).

## Discussion

Our study sought to assess the true situation of centers in Spain with respect to the management of the elderly in general and elderly cancer patients in particular. Furthermore, we collected information about the functioning of oncology departments as regards to the elderly oncological patients and physicians’ needs. Studies have been published in the international literature on this subject [2–5]; however, none of them were based on data from our country. To the best of our knowledge, no prior studies have been carried out in Spain to ascertain medical oncologists’ perception of geriatric oncology and to analyze the Spanish situation related to management of elderly cancer patients; hence, the originality of our paper.

Other efforts have been ongoing for many years in Spain. Molina et al. have worked for years in the field of geriatric oncology [6] and two national, multidisciplinary meetings were held in 2011 and 2012 [7, 8]. To date, three units have been recognized by SIOG (Hospital Universitario Fundación Jiménez Díaz in Madrid, Hospital Duran i Reynals Institut Català d’Oncologia in Barcelona, and Hospital General Virgen de la Luz in Cuenca) [9, 10].

After creating the Geriatric Oncology Working Group on behalf of SEOM, one of the objectives was to determine the Spanish physicians’ needs with respect to elderly patients with cancer.

Unfortunately, participation in completing the survey was low (approximately 12%), if we consider that Spain has at least 1216 medical oncologists (not all of whom are SEOM members). The response rate (12%) of our study was unsatisfactory, yet comparable to those for most surveys of this type. Kurtz et al.’s study [5] reported a response rate of 49.6%, although in that work, perceptions were collected from general practitioners, not from oncologists, as in our survey. If we focus solely on medical oncologists, the mean response rate reported in the literature is not much higher. Monfardini et al. collected 199 answers from only the heads of oncology services [2]. We regret that the personal e-mail (following the website questionnaire) did not improve the response rate among Spanish medical oncologists.

The questionnaire was intentionally straightforward, so that it could be filled in quickly; we therefore did not collect respondents’ characteristics (age, gender, and type of practice and cancer location). A selection bias could not be avoided, as the medical oncologists most highly motivated in geriatric oncology may have been the ones who responded to the survey.

Consequently, greater effort should be made through the Working Group to motivate Spanish medical oncologists. We are unsure if there is a lack of interest in geriatric oncology, if they don’t perceive aging and cancer as problems that affect them, or whether they simply receive so much work via e-mail that answering a questionnaire was perceived to be too time-consuming. We attempted to draft a simplified questionnaire that could be completed in 5–10 min, so as to capture as much participation as possible, albeit at the expense of other relevant information. The website was case-sensitive; as a result, the survey could not be completed twice by the same person.

The first question had to do with managing the elderly in general, not cancer patients in particular. Most centers that answered care for seniors in almost all the departments (internal medicine, palliative care, social work…); hence, we can conclude that Spanish hospitals are well-equipped. While all hospitals in Spain have internal medicine departments, the answer was not a 100% given, that where there is a geriatrics discipline, the latter are in charge of elderly patients. Regrettably, fewer than half have geriatricians (74; 48%). One reason that not all hospitals have a geriatrics department may be because, though the Spanish Society of Geriatrics and Gerontology (SEGG) have 1773 members dedicated to the elderly, not all of them are geriatricians. It has been calculated that there are currently only 420 geriatric specialists in the public healthcare system (850 counting the private system), compared to the close to nine million elderly people living in Spain [10]. It is, therefore, difficult to have a geriatric discipline at all the centers.

When asked if those disciplines also care for elderly patients with cancer (in addition to the oncology department), it is striking to note that, in addition to the scarcity of centers with a geriatric Service, not all of them (50, 32%) dedicate their care time to seniors with cancer.

One hypothesis from our data might be that there is a poor distribution of geriatric departments among Spanish centers and that most of them focus on other, non-oncological diseases affecting seniors.

One reason for this could be that we lack a National Geriatric Oncology Plan in Spain similar to those in France or Belgium [11–14]. The French National Cancer Plans implemented Geriatric Oncology Coordination Units in France specifically to enable all elderly people with cancer in all regions to benefit from specialized care management.

Insofar as the functioning of medical oncology services, only a minority of had a reference figure or coordinator specifically dedicated to older patients, either alone or in close cooperation with geriatricians (14%). This is likely the result of the afore-mentioned lack of a National Plan, as each center is currently doing the best they can without a real coordinator or strategy in place. Therefore, specific protocols (7%) or geriatric assessments (31%) were present at only a few units.

Spain suffers a clear delay in transferring the results and recommendations of geriatric assessment into practice [9]. Regrettably, this is in line with the situation across Europe, where geriatric oncology is largely unknown and geriatric scales are used by a relatively small percentage of medical oncologists.

Of special importance is the fact that most of the physicians participating in the survey were convinced that there is an increase in the presence of elderly cancer patients in clinical practice, because of the need for specific protocols, guidelines, and education. Virtually all of the medical oncologists requested training and education. Based on these data, we have determined that there is a huge gap between theory and practice in our country. If most of the participating physicians agreed that there is a need for geriatric oncology, why is the use of geriatric assessment so low?

All this information regarding the truth of the situation in Spain and the need for a specific approach, guidelines, and training has prompted the Working Group to hold meetings, sessions, and reviews. Since the survey was conducted, the first meeting on geriatric oncology in Spain took place on 15 May in Valencia. It was enthusiastically received and commanded a large turnout. A specific Delphi document of recommendations for geriatric assessment in our country has been written (in press). The Group has contacted the Spanish Society of Geriatrics and Gerontology (SEGG) and signed a document to collaborate with them.

**Fig. 5:**
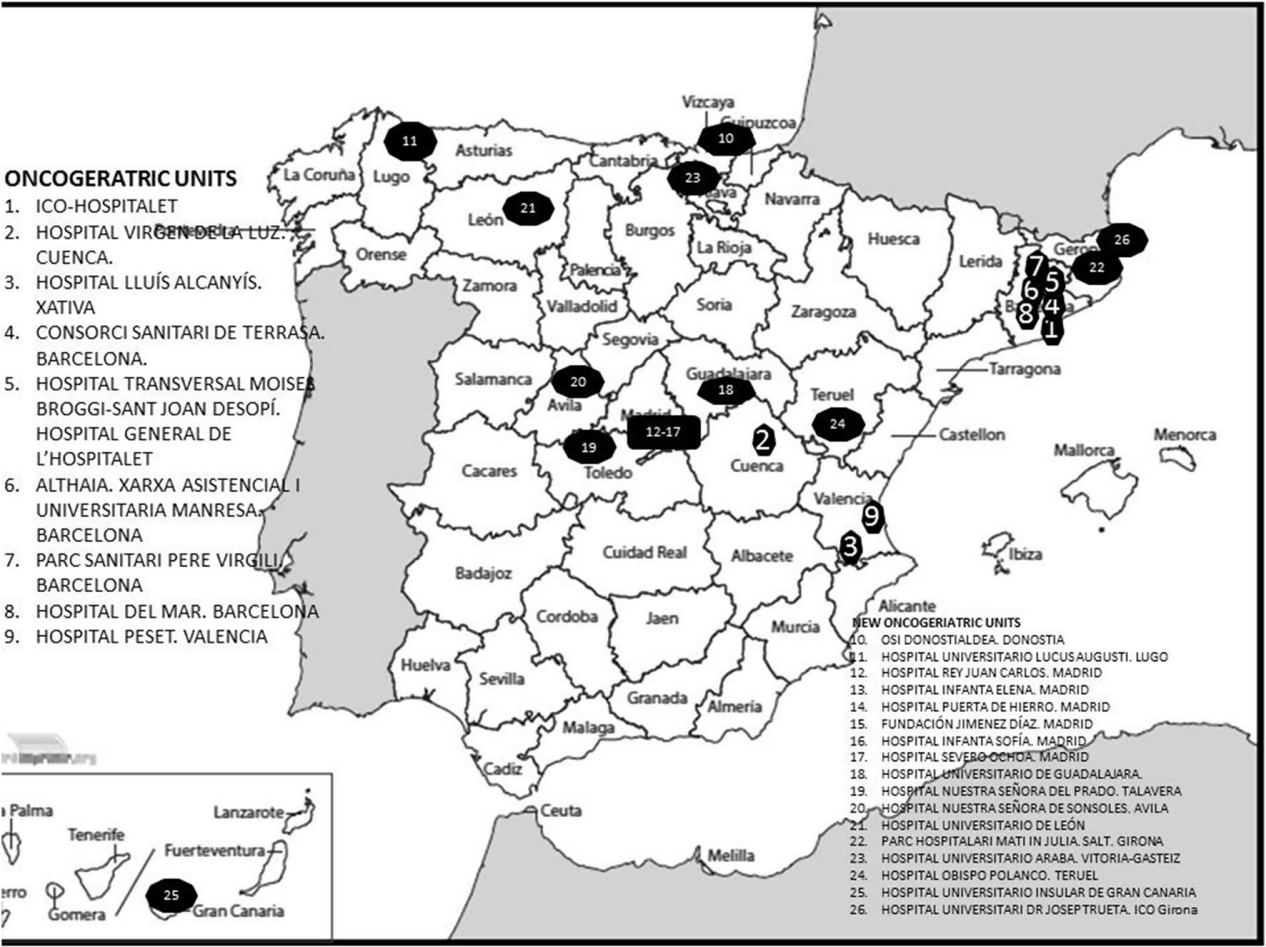
Location of onco-geriatric units in Spain

This work represents progress in that it reviewed the true situation in Spain and, as a result, many geriatric oncology consultations have been held this year (Fig. 5). Many relevant studies about cancer in the elderly will be carried out in our country for the coming years.

We believe that although our work has certain limitations, substantial effort has been made and should be reflected in the international literature. There are several obstacles to implementing geriatric evaluation in our daily clinical practice, such as lack of time, insufficient availability of geriatricians, as well as the lack of a National Plan. Most of the barriers identified were organizational, with high workload, lack of time or funding/staffing as the most cited.

The newly created Working Group has set forth the following missions: to adapt cancer treatment in seniors and make it possible for all the elderly cancer patients to benefit from this geriatric oncology approach; stimulate specific research in geriatric oncology; promote training for health professionals, and to develop information. Routine clinical use of a geriatric screening tool must become more widespread. Lastly, recommendations must be developed for treatment strategies tailored specifically to elderly persons with high-incidence cancers.

To conclude, the original purpose of this study was to assess the interest and feelings of Spanish medical oncologists as regards to geriatric oncology. Our data reflect a heterogeneous manner of dealing with elderly cancer patients in our country. Most of the respondents perceived unmet needs, including training. In short, geriatric oncology in our country has only just begun—humbly, yet unstoppably.
